# Plasticity Induced in the Human Spinal Cord by Focal Muscle Vibration

**DOI:** 10.3389/fneur.2018.00935

**Published:** 2018-11-02

**Authors:** Lorenzo Rocchi, Antonio Suppa, Giorgio Leodori, Claudia Celletti, Filippo Camerota, John Rothwell, Alfredo Berardelli

**Affiliations:** ^1^Department of Clinical and Movement Neurosciences, UCL Queen Square Institute of Neurology, University College London, London, United Kingdom; ^2^Department of Human Neurosciences, University of Rome “Sapienza”, Rome, Italy; ^3^Department of Clinical Neurophysiology, IRCCS Neuromed Institute, Pozzilli, Italy; ^4^Physical Medicine and Rehabilitation Division, Sapienza University of Rome, Rome, Italy

**Keywords:** H-reflex, reciprocal inhibition, muscle vibration, spinal cord, plasticity, somatosensory evoked potentials

## Abstract

The spinal cord spinal cord has in the past been considered a hardwired system which responds to inputs in a stereotyped way. A growing body of data have instead demonstrated its ability to retain information and modify its effector capabilities, showing activity-dependent plasticity. Whereas, plasticity in the spinal cord is well documented after different forms of physical exercise, whether exogenous stimulation can induce similar changes is still a matter of debate. This issue is both of scientific and clinical relevance, since at least one form of stimulation, i.e., focal muscle vibration (fMV), is currently used as a treatment for spasticity. The aim of the present study was to assess whether fMV can induce plasticity at the SC level when applied to different muscles of the upper limb. Changes in different electrophysiological measures, such as H-reflex testing homonymous and heteronymous pathways, reciprocal inhibition and somatosensory evoked potentials were used as outcomes. We found that fMV was able to induce long-term depression-like plasticity in specific spinal cord circuits depending on the muscle vibrated. These findings helped understand the basic mechanisms underlying the effects of fMV and might help to develop more advanced stimulation protocols.

## Introduction

The ability of the spinal cord to retain information and modify its effector capabilities based on activity-dependent changes in the strength of synaptic transmission is well known (1–3). Neural activity produced by experience can have different biological effects, effects including sprouting, long-term potentiation and depression, gene activation, and dendritic modifications (2). Spinal plasticity has been demonstrated in healthy humans after several types of physical exercise, including running (4) and cycling (5–7). A few studies also suggested that it is possible to induce spinal plasticity by means of repetitive electrical stimulation of peripheral nerves (8, 9). This has led to the idea that exogenous stimulation might be used to induce activity-dependent plasticity in the spinal cord in the clinical setting, and possibly be helpful for therapeutical approaches in various neurological disorders (1, 2). At least one form of stimulation, i.e., focal muscle vibration (fMV), has already been implemented as a therapeutical for neurologic conditions such as spasticity (10, 11). Focal muscle vibration (fMV) entails the use of an electromechanical transducer to apply a vibratory stimulus with controllable frequency and displacement over the muscle-tendon complex (12–15). This vibration is well-known to activate afferent somatosensory input fibers (16), however its central mechanisms of action remain somewhat elusive. A body of literature has pointed toward presynaptic changes in the spinal cord, which are reflected by a decrease in the amplitude of the H reflex (HR); this phenomenon is known as post-vibration depression (PVD) (1, 2). However, it has also been demonstrated that fMV can induce plasticity at the cortical level, reflected by changes in excitability and extension of motor maps in the primary motor area (15) and changes in patterns of sensorimotor interaction (17). We then hypothesized that the effects of fMV could be explained by plasticity in the spinal cord, which has not been investigated so far.

Specifically, In the present paper we sought to investigate whether a long session of fMV could induce plasticity in the spinal cord. Duration, frequency and displacement induced by fMV were chosen based on previous protocols which were demonstrated to induce cortical plasticity and to be selective for stimulation of Ia afferent fibers (18–20). Outcome measures included HR recorded from the flexor carpi radialis muscle (FCR) and the three phases of reciprocal inhibition (RI) between FCR and extensor carpi radialis (ECR), which tests different aspects of spinal cord circuitry in detail (21, 22). To better characterize the effects of fMV, we delivered our vibration protocol over FCR as well as over an antagonist muscle (ECR) and over biceps brachialis (BB) and abductor pollicis brevis (APB). To better control for possible confounding due to muscle activation, we verified whether sustained contraction of FCR or ECR alone (without fMV) influences HR amplitude *per se*. Finally, we recorded upper limb somatosensory evoked potentials (SEP) stimulating the median nerve at the elbow to clarify whether fMV was associated to changes in effectiveness of transmission along the somatosensory pathway, and obtained heteronymous HR (HHR) from FCR obtained by stimulation of the ulnar nerve (23–26) to investigate possible post-synaptic effects of our fMV protocol. This information would give important insight into the physiological response of specific spinal cord circuits to a well standardized externally driven stimulation such as fMV and help to further develop new fMV protocols as a therapeutic tool in neurological disorders.

## Materials and methods

### Ethical approval

All procedures were carried out with the adequate understanding and written informed consent of the subjects prior to the experiments. All experimental procedures were conducted in accordance with the Declaration of Helsinki and according to international safety guidelines. Formal approval to conduct the experiments described has been obtained from the human subjects review board of the University of Rome “Sapienza” (IRB approval number 1512) and could be provided upon request.

### Subjects

Nineteen healthy subjects (4 female and 15 male, mean age 31.4 ± 8.4), all right handed (27), were enrolled in the study. Participants had no history of any neuropsychiatric disorders, neurosurgery, or metal or electronic implants and were not on drugs active at the central nervous system level at the time of the experiments.

### Experimental setting

Subjects were comfortably seated in an armchair beside a table in a quiet room. The right arm, tested during the whole study, rested on the table and was firmly secured to ensure a consistent positioning and to avoid stretch of the test muscle. The arm position was also kept constant throughout the experiment by visually inspecting the joint angles and by keeping the distance between the armchair and the table stable throughout the experiments. The right arm was placed with the shoulder in slight abduction (60°), the elbow semi-flexed (110°), the forearm pronated and supported by the arm of the chair while the wrist and the hand were kept in the neutral position.

### Electromyographic recording

Electromyography (EMG) was recorded through 1 cm diameter Ag/AgCl surface electrodes placed over FCR, ECR, and flexor carpi ulnaris (FCU) muscles. The active electrode was placed roughly at the center of the muscle belly, while the reference was placed 2 cm distally to minimize activity from surrounding muscles (28). When recording from BB and APB for the purpose of visual feedback during fMV (see below), the electrodes were arranged in a belly-tendon fashion. Raw signal, sampled at 5 kHz with a CED 1401 A/D laboratory interface (Cambridge Electronic Design, Cambridge, UK), was amplified and filtered (bandwidth 20 Hz− 2 kHz) with a Digitimer D360 (Digitimer Ltd., Welwyn Garden City, Hertfordshire, UK). Data were stored on a laboratory computer for on-line visual display and further off-line analysis (Signal software, Cambridge Electronic Design, Cambridge, UK). To ensure complete target muscle relaxation throughout the experimental sessions we continuously monitored the EMG activity with audio and high-gain visual feedback.

### Peripheral nerve stimulation: H-reflex, reciprocal inhibition, and heteronymous responses

HR was elicited through stimulation of the median nerve in the antecubital fossa through surface electrodes set 2 cm apart. The anode was placed distally to avoid the possibility of anodal block (29). Electric pulses were supplied by two constant current stimulators (DS7A, Digitimer, Welwyn, UK); square wave pulse of 1 ms were used to preferentially stimulate Ia afferents (28). First, a maximal M wave (M_max_) and HR (H_max_) were obtained (30). A stimulation intensity able to elicit a HR of ~50% of its maximum amplitude (H_50_) was used, thus avoiding the possibility that reciprocal Ia inhibition was contaminated by Ib and recurrent inhibitory pathways (30). In all subjects, the intensity used was able to elicit a small M wave, the amplitude of which was checked throughout the experiment to ensure a constant stimulation effectiveness. The radial nerve was stimulated at the spiral groove above the elbow, using square wave electrical pulses of 500 μs duration (30). Ninety percentage of the intensity able to produce an M wave of around 50 μV (ECR_thr_) amplitude was used. A stimulation intensity below motor threshold (MT) was used to selectively stimulate Ia afferents and reduce the possibility of Ib afferent discharges (30). RI at the wrist assesses the interaction between stimulation of the radial nerve, supplying the extensor muscles of the forearm, and the HR produced by stimulation of the median nerve. A reduction in the size of the HR occurs at particular interstimulus intervals (ISI) in normal subjects (21, 22). RI was recorded stimulating, in separate states, the median nerve alone and both median and radial nerves at ISI of −1, 0, 1, 3, 5, 10, 20, 50, 70, 100, and 300 ms, where positive ISI indicates that the stimulus applied over the radial nerve precedes the one on the median nerve (31). A total of 12 trials per state were recorded, in a randomized way, at a frequency of 0.1 Hz to minimize the risk of homosynaptic depression (32). HHR was obtained by a method similar to the one used in previous studies except that stimulation was performed on the ulnar nerve at the elbow rather that at the wrist (25, 26). The basic methodology is the same as that mentioned for HR and 30 responses were averaged due to the lower signal-to-noise ratio. The stimulation used was such as to obtain stable HHR from FCR (between 1.5x and 2x motor threshold of the FCU) (25, 26).

### Somatosensory evoked potentials (SEP) recording

SEP were recorded from Ag-AgCl surface electrodes placed on the Erb point ipsilateral (active electrode) and contralateral to stimulation (reference electrode), over the 6th cervical spinous process (active electrode) and the anterior aspect of the neck (reference electrode), and finally at CP3 (active electrode) and Fz (reference electrode), according to the international 10–20 EEG system (33, 34). Median nerve at the right elbow (antecubital fossa) was stimulated with a constant current stimulator (Digitimer DS7A), with the cathode kept proximal to the anode. Stimulation consisted of square wave pulses given at a frequency of 3 Hz. Signal was recorded from 20 ms before to 100 ms after the electric pulse, digitized with a 5 KHz sampling frequency and band-pass filtered from 3 Hz to 2 KHz (34). Stimulation intensity was set at 200 and 300% of the perceptual somatosensory threshold in two separate blocks, each block consisting in 500 trials. The amplitude of different SEP components was measured and the values were used for the following analyses.

### Focal muscle vibration

FMV was delivered by using a specific device consisting of an electromechanical transducer, a mechanical support, and an electronic control device (CRO®SYSTEM, NEMOCO srl, Italy). The mechanical support allowed the orientation, positioning, and rigid fixation of the transducer in every direction relative to the subject's body. The transducer was positioned perpendicularly over the belly of the target muscle in the various experiments. The mechanical support allowed the compression of soft tissues overlying the muscle-tendon complex determining a 0.2–0.5-mm peak-to-peak sinusoidal displacement at a frequency of 100 Hz (19, 20). These parameters were used to ensure the stimulation of Ia afferents (18) and to avoid tonic vibration reflex (12, 35). The fMV intervention consisted of 3 vibration blocks, each with a duration of 10 min, separated by an interval of 1 min. During fMV, subjects kept a steady contraction of the target muscle at 10% of the maximal force, under visual EMG feedback. We chose to deliver fMV during mild voluntary contraction because it was shown that voluntary muscle activity increases response to vibration, probably through fusimotor co-activation and subsequent increase in spindle discharge (36, 37).

### Experimental design

**Figure 1:**
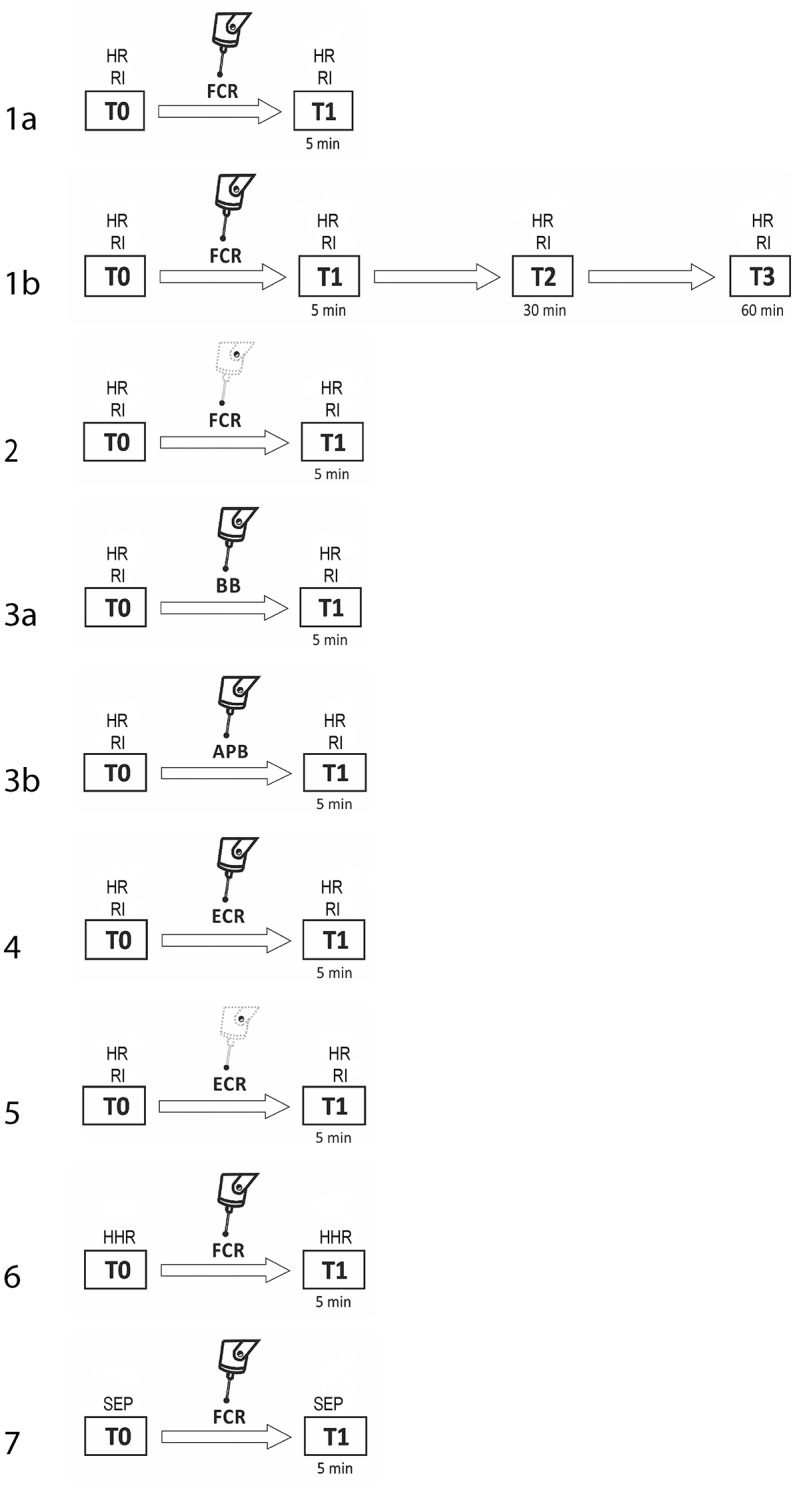
Experimental procedures with regard to RI testing. The number on each row indicates the number of the experiment. Unconditioned HR and RI were investigated before (T0) and 5 min after (T1) fMV applied over FCR (experiment 1a, first line), ECR (experiment 4, sixth line), BB (experiment 3a, fourth line), and APB (experiment 3b, fifth line). The same time course was explored to investigate the effect of voluntary contraction alone of FCR (experiment 2, third line) and ECR (experiment 5, seventh line). In experiment 1b only (second line), the effect of fMV was tested 30 (T2) and 60 (T3) min after fMV applied over FCR. In experiment 6 and 7 HHR and SEP, respectively (eight and ninth line) were recorded before (T0) and 5 min after (T1) fMV.

The procedure consisted of seven experiments, each performed in a separate session. Subjects were pseudo-randomly assigned to participate in the various experimental sessions. For subjects who took part in more than one experiment, at least 1 week elapsed between them. In experiment 1a (fMV applied over the FCR muscle), we evaluated the three phases of RI at baseline (T0) and 5 min after the third block of fMV applied over the FCR muscle (T1) (*n* = 10 subjects, mean age 32.1 ± 6.2). In experiment 1b, we tested the same condition with a long time course (30 min—T2, and at 60 min—T3) after fMV (*n* = 8 subjects, mean age 30.3 ± 5.1). In experiment 2 (contraction of the FCR muscle alone), the same procedure of experiment 1 was applied, except that fMV was not applied (*n* = 10 subjects, mean age 32.7 ± 6.3). In experiment 3 (fMV applied over the BB and APB muscles), we tested RI before and after applying fMV over the ipsilateral BB (experiment 3a) and APB (experiment 3b) (*n* = 10 subjects, mean age 31.6 ± 4.1). In experiment 4 (fMV applied over the ECR muscle), we tested HR and RI before and after fMV applied over ECR, and finally in experiment 5 (contraction of the ECR muscle alone), we examined possible changes in HR and RI following voluntary ECR contraction alone, and thus without fMV (*n* = 10 subjects, mean age 33.4 ± 6.6) (Figure 1). The peak-to-peak amplitude of conditioned and unconditioned HR was measured and the mean amplitude of 12 HR for each state was calculated and used for statistical analysis. Experiment 6 consisted in the recording of HHR as described in the methods section before and 5 min after fMV applied over the right FCR (*n* = 8 subjects, mean age 33.2 ± 7.3). Lastly, in experiment 7 two blocks of SEP, elicited by median nerve stimulation at the elbow, were recorded (using a stimulation intensity of 200 and 300% of the somatosensory threshold, respectively) before and 5 min after fMV over the right FCR (*n* = 8 subjects, mean age 32.1 ± 6.8).

### Statistics

Several three-way mixed ANOVA with “experiment,” “time” (T0, T1) and “ISI” (test, −1, 0, 1, 3, 5, 10, 20, 50, 70, 100, 300) as factors of analysis were performed on conditioned and unconditioned HR to investigate possible effects of fMV on RI at different ISI and different sites of vibration. The factor “experiment” was defined as follows in the different ANOVAs. In ANOVA 1 (experiment 1a and 2) we sought to compare the effect of fMV applied over the FCR and voluntary contraction alone of the same muscle. ANOVA 2 (experiment 2 and 3a) and ANOVA 3 (experiment 2 and 3b) were performed to investigate the effects of fMV applied over BB and APB, respectively, as opposed to voluntary contraction alone of the FCR muscle, while ANOVA 4 (experiment 4 and 5) was used to compare the effect of fMV applied over the ECR and voluntary contraction alone of the same muscle. A final three-way ANOVA (ANOVA 5) on experiments 1a, 3a, 3b, and 4 was performed on conditioned/unconditioned HR ratios to investigate the effect of fMV on inhibition levels. A two-way repeated measures ANOVA with “time” (T0, T1, T2, T3) and “ISI” (test, −1, 0, 1, 3, 5, 10, 20, 50, 70, 100, 300) as factors of analysis was performed to disclose a possible longer duration of the effects of fMV (experiment 1b). Several paired *t*-tests were used to assess the effect of fMV applied over the right FCR on HHR and SEP amplitude (experiment 6 and 7, respectively). Levene's test was used to evaluate possible inhomogeneity of variance among groups and the Shapiro-Wilk test was used to assess normality of distribution. All *p*-values < 0.05 were considered significant. Greenhouse-Geisser correction was used when necessary to correct for non-sphericity (i.e., Mauchly's test < 0.05). Bonferroni correction was applied to interactions and Bonferroni *post-hoc* test was used for all *post-hoc* analyses. Additionally, Observed power (OP) and effect size (η^2^) were calculated for each analysis. All statistical analyses were performed with IBM SPSS v24 (Armonk, NY: IBM Corp).

## Results

**Figure 2:**
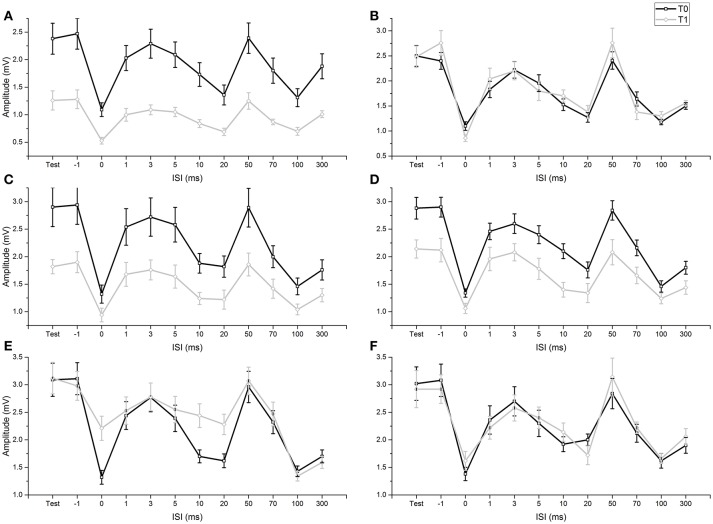
Comparison of conditioned and unconditioned HR amplitude before (T0) and after (T1) fMV. FMV delivered over FCR **(A)**, BB **(C)** and APB **(D)** induced a decrease in the amplitude of unconditioned (test) and conditioned HR at all ISI (all *p*-values < 0.01). By contrast fMV applied over the ECR **(E)** muscle significantly decreased conditioned HR amplitude only at ISI of 0, 10, and 20 ms (all *p*-values < 0.01), while leaving unconditioned HR unchanged. Voluntary contraction alone of FCR **(B)** and ECR **(F)** had no effect. Error bars indicate standard error.

None of the participants experienced any adverse effects during the experiments. Levene's test did not show any significant variance differences across the examined groups (all *p*-values > 0.05) and we did not observe any deviations from normality of distribution (all *p*-values in the Shapiro-Wilk test > 0.05). As sphericity was never violated, it was not necessary to apply the Greenhouse-Geisser correction (*p*-values of Mauchly's tests > 0.05). In our study, M_max_ as well as M wave at intensity for H_50_ remained steady in all subjects and conditions, before and after fMV or voluntary contractions. In addition, before fMV, the unconditioned H_50_ was in a similar range of M_max_ in all subjects. RI was demonstrated at T0 in all experiments comparing the amplitude of HR obtained with a single stimulus (test condition) with the amplitude of HR in the ISI relating to the peak inhibition of the three different RI phases (ISI 0 for the first phase, ISI 20 for the second phase, ISI 100 for the third phase) (all *p*-values < 0.05) (Figure 2). RI was also demonstrated at T1, applying the same comparisons, in experiments 1a, 3a, 3b, thus suggesting that fMV was not able to modulate RI in these cases (see results of ANOVAs for details). In all subjects participating at experiment 6, we recorded clear and stable HHR, and in all participants at experiment 7, we measured clear SEPs at T0 (mean latency: 12.40 ms; mean amplitude: 5.5 uV) and T1 (mean latency: 12.52 ms; mean amplitude: 5.0 uV).

ANOVA 1 (fMV over FCR vs. voluntary FCR contraction alone) showed a non-significant effect of “experiment” [*F*_(1, 18)_ = 3.602, *p* = 0.074] (OP = 0.44, η^2^ = 0.17), a significant effect of “time” [*F*_(1, 18)_ = 23.310, *p* < 0.001] (OP = 0.99, η^2^ = 0.56), “ISI” [*F*_(11, 198)_ = 97.808, *p* < 0.001] (OP = 1, η^2^ = 0.85), and significant interactions of “experiment × time,” [*F*(1,18) = 29.778, *p* < 0.001] (OP = 0.99, η^2^ = 0.85), “experiment × ISI” [*F*_(11, 198)_ = 6.350, *p* < 0.001] (OP = 0.85, η^2^ = 0.26), “time × ISI” [*F*_(11, 198)_ = 4.242, *p* < 0.001] (OP = 0.99, η^2^ = 0.16) and “experiment × time × ISI” [*F*_(11, 198)_ = 8.394, *p* < 0.001] (OP = 1, η^2^ = 0.32). *Post-hoc* comparisons showed that values of test and conditioned HR were smaller after fMV delivered over FCR (all *p*-values < 0.05), while they were not different after voluntary FCR contraction alone (Figure 2).

ANOVA 2 (fMV over BB vs. voluntary FCR contraction alone) showed a non-significant effect of “experiment” [*F*_(1, 18)_ = 0.02, *p* = 0.890] (OP = 0.52, η^2^ = 0.01), a significant effect of “time” [*F*_(1, 18)_ = 29.840, *p* < 0.001] (OP = 0.99, η^2^ = 0.62), “ISI” [*F*_(11, 198)_ = 91.568, *p* < 0.001] (OP = 1, η^2^ = 0.84), and significant interactions of “experiment × time,” [*F*_(1, 18)_ = 40.551, *p* < 0.001] (OP = 1, η^2^ = 0.69), “experiment × ISI” [*F*_(11, 198)_ = 2.151, *p* = 0.019] (OP = 0.92, η^2^ = 0.11), time × ISI” [*F*_(11, 198)_ = 9.869, *p* < 0.001] (OP = 1, η^2^ = 0.20) and “experiment × time × ISI” [*F*_(11, 198)_ = 9.869, *p* < 0.001] (OP = 1, η^2^ = 0.35). *Post-hoc* comparisons showed that values of test and conditioned HR were smaller after fMV delivered over BB (all *p*-values < 0.05), while they were not different after voluntary FCR contraction alone (Figure 2).

ANOVA 3 (fMV over APB vs. voluntary FCR contraction alone) showed a non-significant effect of “experiment” [*F*_(1, 18)_ = 0.512, *p* = 0.484] (OP = 0.10, η^2^ = 0.03), a significant effect of “time” [*F*_(1, 18)_ = 5.574, *p* < 0.001] (OP = 0.61, η^2^ = 0.24), “ISI” [*F*_(11, 198)_ = 128.664, *p* < 0.001] (OP = 1, η^2^ = 0.88), and significant interactions of “experiment × time,” [*F*_(1, 18)_ = 8.583, *p* = 0.009] (OP = 0.791, η^2^ = 0.32), “experiment × ISI” [*F*_(11, 198)_ = 2.745, *p* < 0.003] (OP = 0.97, η^2^ = 0.13), time × ISI” [*F*_(11, 198)_ = 2.880, *p* = 0.002] (OP = 0.98, η^2^ = 0.14) and “experiment × time × ISI” [*F*_(11, 198)_ = 7.074, *p* < 0.001] (OP = 1, η^2^ = 0.28). *Post-hoc* comparisons showed that values of test and conditioned HR were smaller after fMV delivered over APB (all *p*-values < 0.05), while they were not different after voluntary FCR contraction alone (Figure 2).

ANOVA 4 (fMV vs. voluntary ECR contraction alone) showed a significant effect of “experiment” [*F*_(1, 18)_ = 145.851, *p* < 0.001] (OP = 0.05, η^2^ = 0.01), a significant effect of “time” [*F*_(1, 18)_ = 31.053, *p* < 0.001] (OP = 1, η^2^ = 0.63), “ISI” [*F*_(11, 198)_ = 54.243, *p* < 0.001] (OP = 1, η^2^ = 0.75), a significant interaction of “experiment × time,” [*F*_(1, 18)_ = 17.427, *p* < 0.001] (OP = 0.98, η^2^ = 0.49), a non-significant interaction of “experiment × ISI” [*F*_(11, 198)_ = 1.496, *p* = 0.135] (OP = 0.76, η^2^ = 0.77), a significant interaction of time × ISI” [*F*_(11, 198)_ = 11.956, *p* < 0.001] (OP = 1, η^2^ = 0.4) and “experiment × time × ISI” [*F*_(11, 198)_ = 8.176, *p* < 0.001] (OP = 1, η^2^ = 0.31). *Post-hoc* comparisons showed that fMV applied over ECR induced an increase of conditioned HR at ISI of 0, 10 and 20 ms (all *p*-values < 0.05), while no significant change in conditioned and unconditioned HR was observed after voluntary ECR contraction alone (Figure 2).

ANOVA 5 (fMV over FCR, BB, APB and ECR), performed on conditioned/unconditioned HR ratios, showed a non-significant effect of “experiment” [*F*_(3, 36)_ = 0.66, *p* = 0.580] (OP = 0.05, η^2^ = 0.01), a non-significant effect of “time” [*F*_(1, 36)_ = 1.06, *p* = 0.311] (OP = 1, η^2^ = 0.63), a significant effect of “ISI” [*F*_(10, 360)_ = 312.978, *p* < 0.001] (OP = 1, η^2^ = 0.75), a non-significant interactions of “experiment × time,” [*F*_(3, 36)_ = 0.418, *p* = 0.741] (OP = 0.98, η^2^ = 0.49), and a significant interactions of “experiment × ISI” [*F*_(10, 360)_ = 5.53, *p* < 0.001] (OP = 0.77, η^2^ = 0.07), time × ISI” [*F*_(10, 360)_ = 4.70, *p* < 0.001] (OP = 1, η^2^ = 0.40) and “experiment × time × ISI” [*F*_(30, 360)_ = 4.30, *p* < 0.001] (OP = 1, η^2^ = 0.40). *Post-hoc* comparisons showed that fMV applied over ECR induced a decrease in inhibition at ISI of 0, 10, and 20 ms (all *p*-values < 0.05), while no significant change inhibition was observed after fMV over FCR, BB, or ABP.

**Figure 3:**
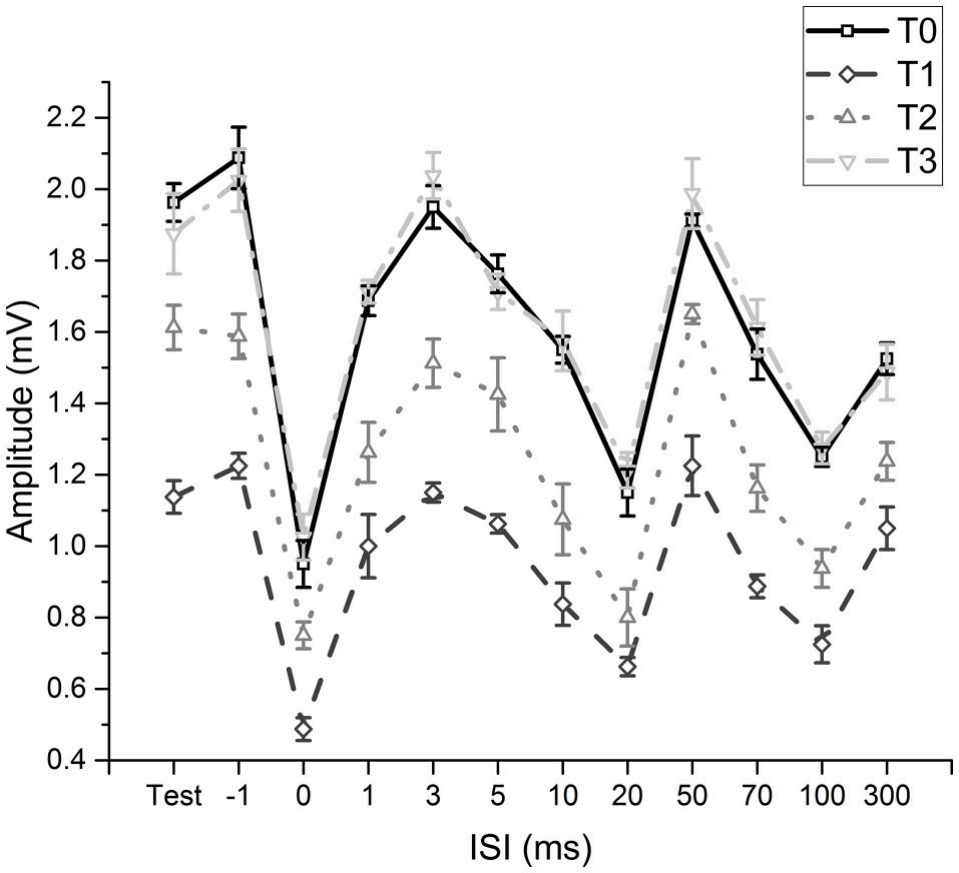
Time-course of effects induced by fMV applied over the FCR muscle. Compared to baseline (T0) fMV decreased unconditioned (test) and conditioned HR amplitude at all ISI tested after 5 min (T1) and 30 min (T2), while HR amplitude returned to values similar to baseline after 60' (T3) (all *p*-values < 0.05). Error bars indicate standard error.

The two-way repeated measures ANOVA (fMV over FCR with longer time course) showed a significant effect of “time” [*F*_(3, 11)_ = 230.234; *p* < 0.001] (OP = 0.99, η^2^ = 0.56) and “ISI” [*F*_(11, 77)_ = 199.926; *p* < 0.001] (OP = 1, η^2^ = 0.85) and a significant interaction of “time × ISI” [*F*_(33, 231)_ = 5.405; *p* < 0.001] (OP = 0.99, η^2^ = 0.19). *Post hoc* analyses showed that after fMV applied on FCR the amplitude of HR decreased at all ISI, as in experiment 1 (all *p*-values < 0.05); the decrease was still significant for all ISI at T2 (all *p*-values < 0.05), while the values returned at baseline at T3 (all *p*-values > 0.05) (Figure 3).

**Figure 4:**
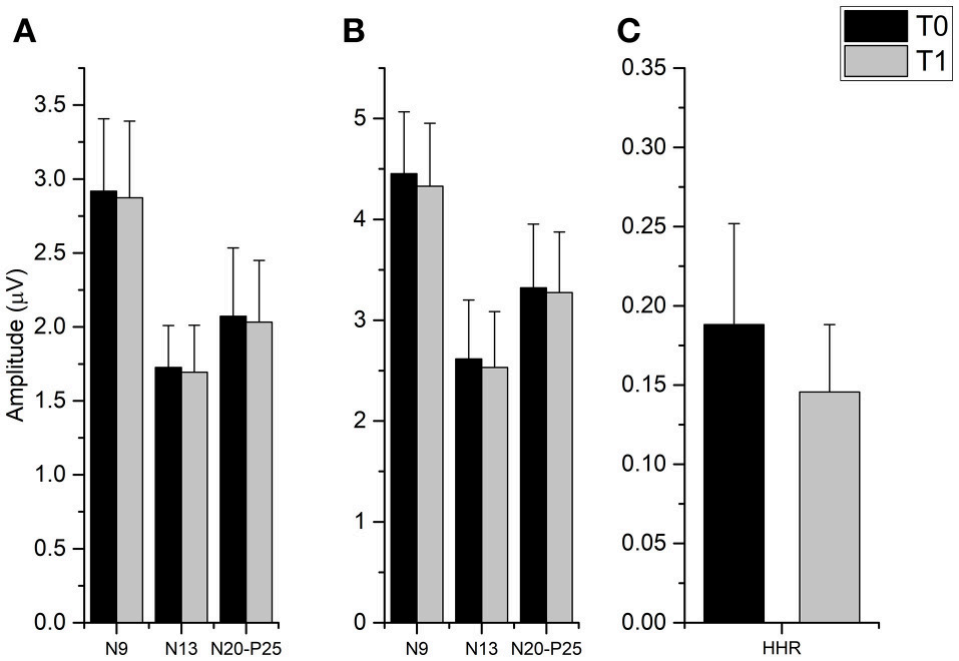
Effect of fMV applied over the FCR muscle on SEP and HHR. fMV has no significant effect on SEP obtained by stimulation the ipsilateral median nerve at the elbow either at 200% **(A)** and 300% **(B)** of the somatosensory threshold. By contrast, fMV induced a significant decrease in the amplitude of HHR (*p* = 0.014) **(C)**. Error bars indicate standard error.

The *t*-tests done on SEP components amplitude did not disclose any significant differences from T0 and T1 in all cortical and subcortical components (all *p*-values > 0.05), while it was found that fMV significantly decreased HHR amplitude [*t*_(7)_ = 3.245, *p* = 0.014) (Figure 4).

## Discussion

In the present study, we found that fMV applied over FCR induced a long-term decrease of HR without modifying the three phases of RI. Similar changes, although of smaller entity, were observed after fMV over BB and APB. Differently, fMV over ECR lead to long-term decrease in first and second but not third phase of RI, without affecting HR. Differently from fMV, sustained muscle contractions of FCR or ECR alone left HR and the three phases of RI unchanged. Finally, fMV over FCR decreased the amplitude of HHR, while leaving SEP components amplitude unchanged. These findings provide evidence that fMV can induce long-term changes in the excitability of specific spinal circuits depending on the vibrated muscle, and suggest that these changes are at least partly due a decreased post-synaptic excitability of spinal motoneuronal pools.

The first finding of this study is that fMV applied over FCR induced a long-term decrease of HR amplitude, a phenomenon termed post-vibration depression (PVD), leaving RI inhibitory level unmodified. The effect was quite strong, with a η^2^ of 0.85 for the interaction of “experiment × time.” This is in line with previous studies reporting a short-term decrease in the amplitude of soleus HR after vibration of Achilles tendon (38) and ankle flexors (39). Given that fMV is known to activate Ia afferents (13, 14, 18), this finding suggests that transmission between Ia afferents and spinal motor neurons (MNs) was impaired at some level. Several mechanisms might explain the long-term changes in HR after fMV (PVD). The observation that sustained voluntary muscle contractions of the FCR left HR unchanged strongly supports that the HR changes we found were crucially related to the application of FCR-fMV. Dindar and Verrier (40) found that fMV applied over anterior muscles of the leg inhibited HR elicited from tibial nerve, implying that PVD was a consequence of post-synaptic non-reciprocal group I inhibition due to activation of muscle spindles in antagonistic muscles caused by a spread of vibration. However, since here fMV applied on ECR did not decrease HR amplitude (see below) our results likely reflect different mechanisms. Hyperpolarization of Ia afferents has been considered as one of the mechanisms mediating PVD (41, 42). However, in the present study, experiment 7 showed that SEP amplitude remained unchanged after fMV, thus suggesting that afferent activity of Ia fibers was intact, as already found by Abbruzzese et al. (16). Moreover, the observation that the third phase of RI was not affected by ECR-fMV (see below) makes it likely that mechanism other than hyperpolarization of Ia afferents must be operating. A purely presynaptic mechanism, such as transmitter depletion from Ia presynaptic terminals, has been often advocated as the main explanation to PVD (16, 43). When we tested the effect of fMV on HHR mediated by heteronymous monosynaptic Ia afferents to FCR (experiment 6), we found that HHR decreased in amplitude suggesting that excitability changes at post-synaptic level probably accounted for part of the observed PVD. Since we were able to record clear and stable HHR in all participants at experiment 6, we suggest that methodological factors might contribute to explain the apparent inconsistency when comparing our findings with those of previous studies demonstrating heteronymous monosynaptic Ia connections to FCR-MNs originating only from intrinsic hand muscles and not from FCU (23–26). Moreover, our experimental approach (ulnar nerve stimulation at the elbow) might have also implied the activation of heteronymous monosynaptic Ia afferents to FCR originating from ulnar nerve-innervated intrinsic hand muscles (23–26).

**Figure 5:**
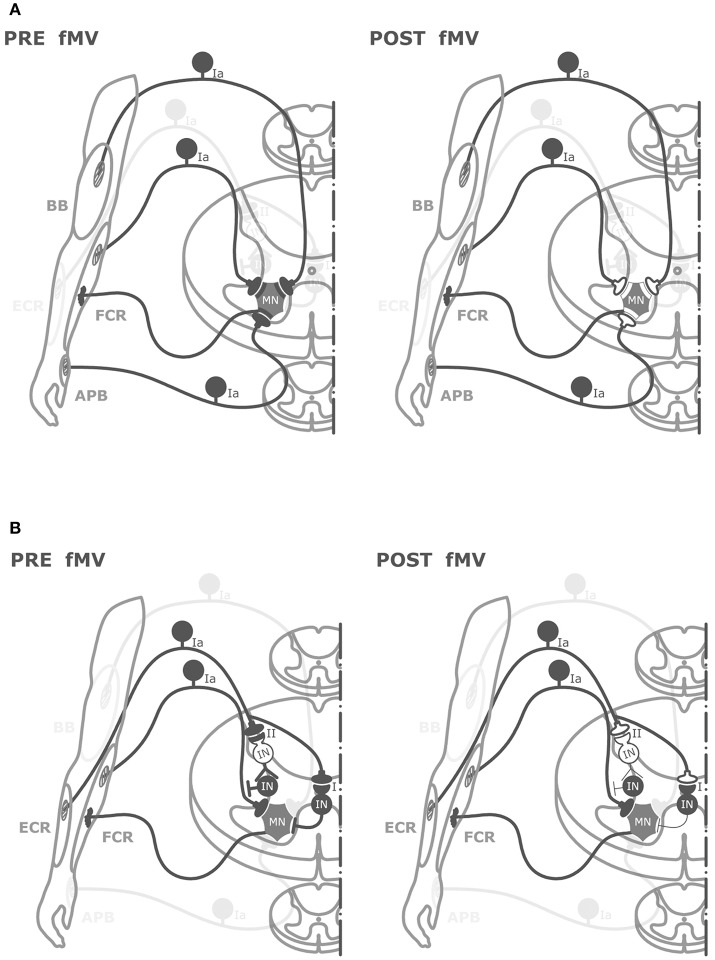
Graphical representation of putative spinal circuits responsible for fMV-induced after effects on HR and RI. **(A)** Spinal circuits modulated by FCR-, BB-, and APB-fMV. Ia afferents from FCR, BB, and APB muscle spindles have homonymous and heteronymous connections (black synaptic boutons) with α-motoneurons (MNs) innervating the FCR muscle. fMV applied over FCR, BB and APB produces a long-term decrease in HR amplitude through a synaptic LTD-like mechanism (white synaptic boutons). **(B)** Spinal circuits modulated by fMV applied over the ECR muscle. fMV induces a form of long-term inhibitory plasticity at the synapse between Ia afferents from ECR and Ia inhibitory interneurons (black circle) mediating the first phase of RI (I), and between Ia afferent and the excitatory interneuron (white circle) in the proximal part of the chain responsible for the second phase of RI (II).

The hypothesis we favor to explain the effect of fMV over the FCR on HR is that FCR-fMV induced long-term decrease of HR amplitude by long-term depression-like (LTD-like) plasticity at the synapse between Ia afferents and MN (Figure 5). LTD is characterized by activity-driven, enduring reduction in synaptic efficacy induced by prolonged stimulation (44). LTD entails pre- and post-synaptic mechanisms such as a reduction in neurotransmitter release (45) and a number of α-amino-3-hydroxy-5-methyl-4-isoxazolepropionic acid (AMPA) receptors modifications such as redistribution (46), dephosphorylation and internalization (45), and decrease in conductance due to phosphorylation (46). The reason why we were able to induce post-synaptic changes might be related to the long vibration time and the relatively high frequency used (100 Hz) compared to other studies where presynaptic changes only were found (16, 43). It was already demonstrated that PVD tends to be longer with increasing frequency and duration of fMV (16). It is known that discharge of Ia afferents can be driven in a frequency-locked way by vibration applied up to 500 Hz (13, 18). Thus, it is plausible that the higher frequency used here was more effective in driving Ia heteronymus afferents, possibly being able to induce LTD-like effects. It might be argued that our findings do not fit with the general rule that in the central nervous system, LTD emerges following low-frequency rather than high-frequency stimulation like that applied in our study (100 Hz). However, over the last two decades, this general principle has been largely questioned since a growing number of experimental studies have demonstrated that synapses can dynamically and flexibly express LTP or LTD in response to specific changes in pre-synaptic activity at the time of intervention, by metaplasticity mechanisms (47, 48). Metaplasticity is a higher-order form of synaptic plasticity implying that the threshold for activity-dependent synaptic plasticity is dynamic, and changes as a function of the integrated prior activity of the post-synaptic neuron (47, 48). Metaplasticity often operates with homeostatic mechanisms as predicted by the Bienenstock-Cooper-Munroe (BCM) theory thus preventing uncontrolled forms of synaptic plasticity by stabilizing synaptic transmission within a physiologically meaningful range. Accordingly, in our study, we speculate that muscle contraction during fMV would have increased post-synaptic activity in spinal MNs thus promoting the likelihood for LTD instead of LTP after high-frequency fMV, through homeostatic metaplasticity mechanisms (47, 48).

Another finding in this study is that fMV over FCR left the three phases of RI unchanged. The first phase of RI occurring at short ISIs between −1 and 2–3 ms, is known to be mediated by a spinal disynaptic inhibitory pathway (21) including group Ia interneurons responsible for reciprocal inhibition. Differently, the second phase of RI occurs at longer ISIs ranging from 5 to 30 ms and involves presynaptic inhibition of Ia terminals (22) by interneurons responsible for primary afferent depolarization (PAD). Finally, the third phase of RI is less characterized, but it might reflect a mixture of presynaptic inhibition and trans-cortical mechanisms (31). Although RI was reported to increase with a smaller HR (49), this effect is probably true only when HR is obtained in a wide range of amplitudes (50). Since we investigated the three phases of RI only in a small part of the HR recruitment curve (around 50%), we are confident that the observed changes in HR amplitude at ISI corresponding to the three phases of RI are independent from changes in unconditioned HR.

Another finding in this study is the long-term decrease in HR amplitude when fMV was applied over BB and APB. The effect size was smaller compared to fMV applied over FCR; η^2^ for the interaction of “experiment × time” was 0.69 and 0.32 for and APB, respectively. It can be hypothesized that this effect is mediated by heteronymous connections between muscles of the same limb (Figure 5). Heteronymous Ia connections have been studied in the human upper limb, where they likely assist in the stability of the shoulder girdle and provide support to the hand during manipulatory movements (25). In particular, heteronymous Ia connections from wrist flexors to thenar muscles and BB have been demonstrated (23, 24, 51). Thus, in the present paper, it is likely that fMV applied over BB or APB induced a long-term decrease of HR amplitude through depression of heteronymous Ia connection between these two target muscles and FCR. Our results are apparently in contrast with those of Abbruzzese et al. (16), who found that fMV applied over soleus muscle did not modify the strength of heteronymous inputs from femoral nerve to soleus muscle. This difference, again, might be accounted for by the higher vibration frequency and longer fMV duration used here (see above). A final comment is that the long-term decrease in FCR-HR amplitude following fMV of BB and APB further exclude the hypothesis of fMV-induced activity-dependent hyperpolarization of Ia fibers (pre-synaptic effect) since during fMV of the BB or APB muscles, the Ia-afferents from these muscles are activated, whereas those from the FCR remain quiet. Hence, our findings overall again point to a post-synaptic effect of fMV on FCR MNs.

The last new finding of this study was that fMV applied over ECR caused a long-term decrease of inhibition in the first and second phase but not in the third phase of RI, while HR did not change. Here η^2^ for “experiment × time × ISI” interaction was 0.40. The finding that sustained muscle contractions (without fMV) of the ECR did not affect RI (nor HR) supports the hypothesis that fMV over the ECR is crucially required for eliciting long-term changes of RI. Moreover, the different pattern of effects compared to FCR-fMV suggests that a spread of vibration was not involved as other authors proposed (39). The first phase of RI is considered to be due to activation of a single Ia inhibitory interneuron from Ia afferents originating from spindles in one antagonist muscle (21, 22). Our fMV protocol might then have induced LTD-like plasticity at the synapse between Ia afferents from ECR and group Ia inhibitory interneurons projecting to FCR MNs, thus decreasing the effectiveness of the first phase of RI which is physiologically responsible for reciprocal inhibition (Figure 5). Our interpretation is in contrast with those of Wargon et al. (52) who found that the first phase of RI between FCR and ECR was left unchanged by fMV applied over FCR. Assuming that fMV hyperpolarizes Ia afferents as described before (41), they speculated that the first phase of RI remained unchanged because differently from the lower limb (reciprocal inhibition between soleus and tibialis anterior muscles), the first phase of RI in the upper limb is mediated mostly by Ib afferents at the wrist level (52). However, our observation that SEP were not changed by fMV, as well as a similar finding in a previous study where no reduction in afferent volleys was found after fMV (16) argue against this interpretation. The second phase of RI is thought to be due to Ia afferent depolarization by axo-axonal inhibitory synapses that reduce the size of the presynaptic impulse and decrease the release of excitatory transmitters (22, 53). The pathway responsible for this presynaptic inhibition is likely polysynaptic and involves at least one excitatory interneuron at the beginning of the chain activated by Ia afferents. This pathway is physiologically responsible also for PAD (28, 54). We thus hypothesize that fMV applied over ECR induced LTD-like plasticity between Ia afferents and excitatory interneurons in the proximal part of the chain responsible for presynaptic inhibition, inducing a long-term decrease of RI at an ISI of 10–20 ms. The third phase of RI, which some authors speculate might reflect a mixture of presynaptic inhibition and trans-cortical mechanisms (31) was not modified by fMV on ECR. This finding further supports our hypothesis that fMV induced LTD-like plasticity in the spinal cord without influencing the excitability of long-loop inhibitory connections between the spinal cord and supra-spinal centers (31). Overall the present findings support our hypothesis that fMV is able to induce a form of LTD-like plasticity at the level of spinal cord (Figure 5). We cannot fully exclude however, that mechanisms other than LTD-like plasticity including fMV-induced hyperpolarization of Ia afferents (41) also contributed to our findings.

To sum up, the present data suggest that fMV induces LTD-like plasticity in specific spinal cord circuits depending on the muscle vibrated. Our findings expand the current knowledge about the fMV effects and demonstrate that HR and RI can be useful tools to assess long-term plasticity in the spinal cord. In this view, HR and RI could be used to study the effects of fMV in patients which might benefit from it (11, 55). Moreover, the present data suggest that fMV might be used to modulate heteronymous Ia connections in humans, with potential applications in neurological conditions in which abnormal heteronymous Ia connections are linked to spasticity (56).

## Author contributions

LR conception and design of the work, acquisition, analysis and interpretation of data, drafting the work, AS conception and design of the work, acquisition and interpretation of data, drafting the work, GL, CC, and FC acquisition and interpretation of data, drafting the work, JR and AB conception of the work, interpretation of data, revision of the work.

### Conflict of interest statement

The authors declare that the research was conducted in the absence of any commercial or financial relationships that could be construed as a potential conflict of interest.

## Abbreviations

APB: abductor pollicis brevis
BB: biceps brachialis
CMAP: compound muscle action potential
ECR: extensor carpi radialis
EMG: electromyographic
FCR: flexor carpi radialis
fMV: focal muscle vibration
HR: H-reflex
ISI: interstimulus interval
MN: motor neurons
MT: motor threshold
PAD: primary afferent depolarization
RI: reciprocal inhibition.
