# Corrigendum: Plasticity Induced in the Human Spinal Cord by Focal Muscle Vibration

**DOI:** 10.3389/fneur.2018.01170

**Published:** 2019-01-08

**Authors:** Lorenzo Rocchi, Antonio Suppa, Giorgio Leodori, Claudia Celletti, Filippo Camerota, John Rothwell, Alfredo Berardelli

**Affiliations:** ^1^Department of Clinical and Movement Neurosciences, UCL Queen Square Institute of Neurology, University College London, London, United Kingdom; ^2^Department of Human Neurosciences, University of Rome “Sapienza”, Rome, Italy; ^3^Department of Clinical Neurophysiology, IRCCS Neuromed Institute, Pozzilli, Italy; ^4^Physical Medicine and Rehabilitation Division, Sapienza University of Rome, Rome, Italy

**Keywords:** H-reflex, reciprocal inhibition, muscle vibration, spinal cord, plasticity, somatosensory evoked potentials

In the published article, there was an error regarding the affiliation for Giorgio Leodori. As well as having affiliation two, “Department of Human Neurosciences, University of Rome “Sapienza”, Rome, Italy”, he should also have affiliation three, “Department of Clinical Neurophysiology, IRCCS Neuromed Institute, Pozzilli, Italy.”

The authors apologize for this error and state that this does not change the scientific conclusions of the article in any way. The original article has been updated.

